# Intra-abdominal Rupture of a Live Cervical Pregnancy with Placenta Accreta but Without Vaginal Bleeding

**DOI:** 10.5811/cpcem.2017.10.32029

**Published:** 2018-03-14

**Authors:** Asma Tariq, Maria O’Rourke, Steven J. Carstens, Vicken Y. Totten

**Affiliations:** Kaweah Delta Health Care District, Department of Emergency Medicine, Visalia, California

## Abstract

We describe an unusual ruptured ectopic pregnancy. The unique features of the case include abdominal pain without vaginal bleeding; cervical implantation and a placenta accreta; and the late presentation at 16 weeks of gestation without prior symptoms. Both the initial point-of-care ultrasound and the formal ultrasound were interpreted as showing an intrauterine pregnancy. The clinical presentation was misleading; the correct diagnosis was made by magnetic resonance imaging. We show the ultrasonic images. We discuss cervical ectopic pregnancies, their diagnosis and management. The woman survived but required emergency hysterectomy and many units of blood.

## INTRODUCTION

We report an unusual case of a ruptured live cervical ectopic pregnancy, which presented without vaginal bleeding. Unrecognized rupture of an ectopic pregnancy can be catastrophic; the most common presentation is vaginal bleeding. Cervical pregnancies are especially prone to visible bleeding, yet our patient presented with abdominal pain alone. Unique features of this case included the presentation without vaginal bleeding; a live fetus of 16 weeks gestation (second trimester) and both a point-of-care ultrasound (POCUS) and a formal ultrasound that were interpreted as demonstrating a live fetus at 16 weeks; and a definitive diagnosis suggested by magnetic resonance imaging (MRI), which disputed the ultrasonic findings. This combination of circumstances is exceedingly rare: no other case has been reported in which MRI was definitive, yet the possibility must be kept in mind by emergency physicians. We describe the incidence and predisposing factors for cervical pregnancy and discuss diagnostic modalities and treatment options.

## CASE REPORT

A 21-year-old-female presented to the emergency department (ED) with sudden onset abdominal pain and vomiting that started one hour prior to arrival in the ED. The pain was diffuse but worse in the epigastrium and the right lower quadrant (RLQ); it radiated into the chest and neck. She reported dysuria and a subjective fever, but denied vaginal bleeding. Her last menstrual period was two months prior. She denied current pregnancy or previous sexually transmitted diseases. Just over a year earlier, she had delivered a single, live-term infant by Cesarean section. She denied recent dietary changes or history of gallstones.

On presentation, the patient was in moderate distress and was alert and cooperative. Initial vital signs were pulse 65 beats/minute; respiratory rate 24 breaths/minute; and blood pressure of 111/50 millimeters of mercury. She was afebrile. Oxygen saturation was 99%. Cardiac and lung examinations were normal. The abdominal exam revealed diffuse tenderness to palpation with involuntary guarding and rebound in the right upper quadrant (RUQ), left upper quadrant (LUQ), and the RLQ. She refused a pelvic exam due to discomfort, and preferred to lie on her right side.

The differential diagnosis of peritonitis in this patient included acute pancreatitis, perforated peptic ulcer, appendicitis or cholecystitis with perforation, ovarian cyst rupture and vascular catastrophe including ectopic pregnancy. Her urine pregnancy test was positive, changing the differential diagnosis. The quantitative human chorionic gonadotropin (HCG) level was within the discriminatory zone at 31239 mIU/ml; lipase was negative; initial complete blood count (CBC) revealed a white cell count of 19.25 x 10 9 / L and hemoglobin of 11.6 g/dl.

**Image 1:**
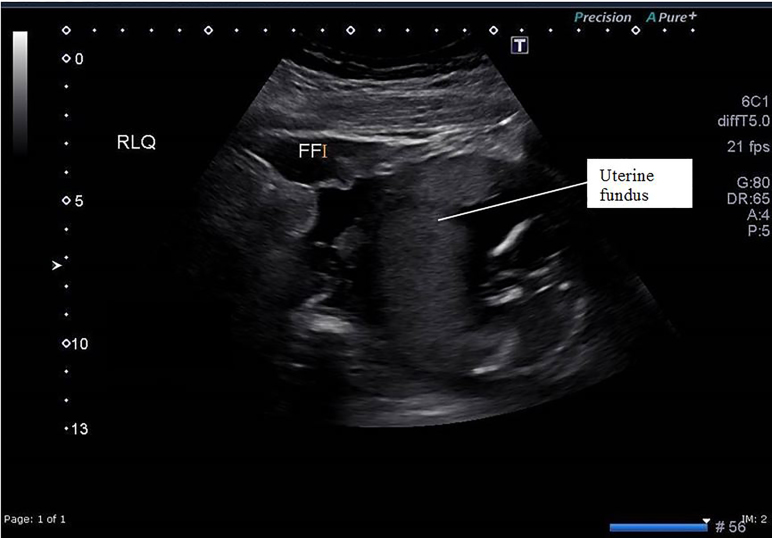
Sagittal view of the uterus showing viable intrauterine pregnancy at 16 weeks and a large amount of free fluid (FF).

**Image 2:**
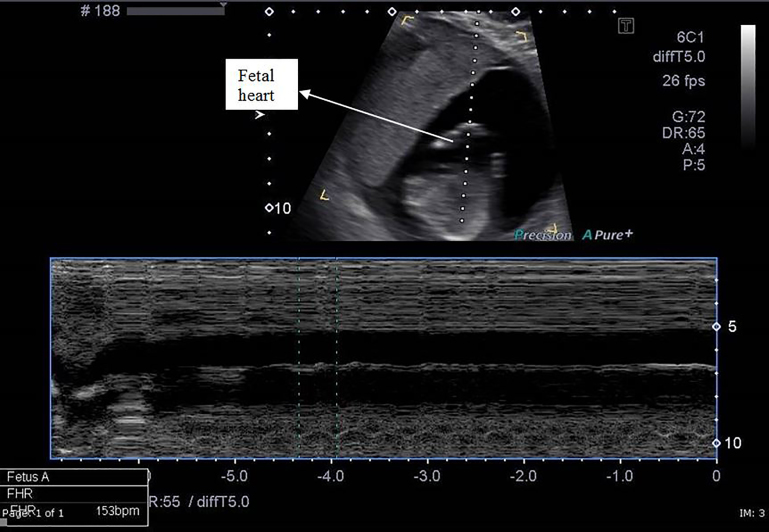
M-mode through the fetal heart demonstrating a rate of 153 beats / minute in cervical ectopic pregnancy.

Two large-bore intravenous (IV) lines were placed, and fluids given. A POCUS in the ED showed what appeared to be a live intrauterine pregnancy, with a large amount of free fluid around the spleen. There was also free fluid in the RUQ and RLQ. A formal ultrasonographic examination was interpreted as showing a 16-week, live, intrauterine pregnancy with an anterior placenta, as well as a large amount of free fluid around the spleen and in the RLQ (Image 1 and 2.)

Therefore, the differential changed to an acute abdomen in the setting of what was presumed to be a live intrauterine pregnancy. Both an obstetrician and a general surgeon were emergently consulted. The differential diagnosis now included appendicitis, a ruptured ovarian cyst, or possibly a ruptured cervical ectopic pregnancy, in spite of an ultrasound read as intrauterine pregnancy. The consultants requested a MRI scan for clarification. The patient was deemed stable enough to delay operation, so this was done.

The MRI of the abdomen and pelvis was interpreted as revealing a low fetal implantation near the cervix concerning for cervical ectopic pregnancy. The majority of the uterine body was seen well above the location of the fetus. An irregularity of the anterior right aspect of the cervix suggested possible extra-uterine placental extension. The free fluid throughout the abdomen demonstrated an increased T1 signal (T1 is an MRI sequence wherein degraded blood and sub-acute hemorrhage produce a signal of increased intensity), further suggesting that the cervical pregnancy had ruptured into the abdomen rather than bleeding vaginally.

That the intra-abdominal fluid was an acute, large volume, intra-abdominal blood loss was supported by a drop in hemoglobin over a few hours from 11.6 g/dl at presentation to 9.7 g/dl. Therefore, three units of packed red blood cells (PRBCs) were prepared and transfusion started. The obstetrician promptly took the patient to the operating room.

Prior to operating, the possibility of emergent hysterectomy was raised with the patient in the event that hemorrhage could not otherwise be controlled. She agreed. Under anesthesia, the cervix was found to be closed. There was a normal vaginal mucosa and no transvaginal bleeding. On bimanual exam the uterus was noted to be very soft. The exploratory laparotomy revealed a ruptured cervical pregnancy with a placenta accreta protruding out of the right uterine wall and bleeding heavily.

The fetus and placenta were removed via a classic vertical uterine incision. Unfortunately, it was not possible to control hemorrhage at the site of the placenta accreta extrusion. The cervix and uterine tissues were very thin and friable. A hysterectomy was performed to stop the ongoing blood loss. At the end of the operation, the patient’s hemoglobin had dropped to 6.4g/dl. The patient received an additional two units PRBCs and did well post-operatively. Her hospital course was unremarkable and she was discharged home on post-operative day three.

**CPC-EM Capsule**

What do we already know about this clinical entity?

Ectopic pregnancies should always be considered in women of childbearing age who have abdominal pain, even without vaginal bleeding. Ectopic pregnancies can implant anywhere.

What makes this presentation of disease reportable?

Several features of this case are unique: placenta accreta with cervical pregnancy bleeding only intraabdominally and 2 ultrasounds reported as IUPs. MRI was diagnostic.

What is the major learning point?

Ultrasound, even transvaginal, may be misleading. When the clinical situation warrants it, consider MRI.

How might this improve emergency medicine practice?

MRI is rarely needed to localize a pregnancy, but is a modality to keep in mind. Also warn such a patient of the possibility of hysterectomy.

## DISCUSSION

Cervical pregnancy is a rare and dangerous form of ectopic pregnancy that results from implantation of the zygote below the internal os. Cervical pregnancies have an incidence of approximately 1:9,000 of all pregnancies^1^ and account for 0.15% of all ectopic pregnancies.^2^ Risk factors for cervical implantation include a prior history of cervical dilatation, uterine curettage, or Cesarean section^3^,^4^ Cervical ectopic pregnancies may also be more prevalent after assisted reproduction, occurring in an estimated 0.1% of in vitro fertilizations.^5^,^6^

Like all other ectopic pregnancies, cervical pregnancies have the potential for massive hemorrhage causing death. Mortality rates in the earliest reported cases were 40% to 45%. However, since 1954 no maternal deaths as a result of cervical pregnancy have been documented in the medical literature.^7^–^10^ This reduction in mortality is partly attributable to earlier (ultrasonic) recognition, and also to aggressive resuscitation, newer transfusion therapies and improved surgical techniques.

Vaginal bleeding is the most common presenting symptom of cervical pregnancy.^11^ Unexpectedly, our patient had no vaginal bleeding. Other clinical criteria of cervical pregnancy involve closed internal os, partially open external os, products of conception confined within the endocervix, and a softened and disproportionately enlarged cervix.^12^–^14^ Early clinical suspicion and recognition of cervical ectopic pregnancy is critical to decreasing potentially fatal hemorrhage. Timely detection also allows an attempt at conservative management strategies.

The methods used to diagnose cervical ectopic pregnancy have changed over time. In the past, cervical ectopic pregnancy was diagnosed intra-operatively in the presence of extensive hemorrhage at the time of uterine curettage. The advent of transvaginal ultrasound has greatly enhanced diagnostic options. However, as our case reported, sometimes even transvaginal ultrasound is insufficient to make the diagnosis. Together with a clinical suspicion, rapid assays of serum HCG, and advanced imaging (in our case, MRI), the diagnosis of cervical ectopic pregnancy can be made much earlier than was historically possible.^3^–^16^.

There are strict sonographic criteria for the diagnosis of cervical ectopic pregnancy.^2^, ^13^, ^17^ The criteria are as follows: (1) intra-cervical localization of the ectopic gestation; (2) closed internal os; (3) trophoblastic invasion in the endocervical tissue; (4) empty uterine cavity; (5) hourglass-shaped uterus; (6) intra-cervical peritrophoblastic blood flow; and (7) diffuse amorphous intrauterine echoes.

Even with the widespread availability of ultrasound, clinical recognition of cervical ectopic pregnancy remains difficult. Ultrasonic diagnosis is correct in 82% of cases.^18^ Culdocentesis is now rarely used for the diagnosis of ectopic pregnancy. It has largely been replaced by ultrasonic imaging of free fluid in the cul de sac (pouch of Douglas). However, in those facilities lacking ultrasound, or if the clinical presentation is confusing, aspirating non-clotting blood from the pouch of Douglas may provide useful information and influence the choice of therapy. Blood aspirated from the pouch of Douglas that does not clot on standing is suggestive of an ectopic pregnancy.^19^, ^20^ In our patient, the MRI clinched the diagnosis of ruptured cervical pregnancy; culdocentesis was not performed.

Initial evaluation after a physical examination should include a CBC, blood type and screen, and a quantitative β-hCG measurement followed by transvaginal ultrasound. This can help determine the approximate gestational age and guide the physician in choosing the appropriate therapy. Conservative management is the therapy of choice when the diagnosis is early and before complications arise.

The choice of surgical management vs. medical treatment of cervical pregnancies is dictated by the length of the gestation and the hemodynamic stability of the patient. The main goal of conservative therapy is to preserve the patient’s reproductive capability.^21^ Medical management of ectopic pregnancies usually includes a cytotoxic agent such as methotrexate, and is most effective when the conceptus is small. Pregnancies of greater than six weeks duration generally require induction of fetal death (usually with potassium chloride) or high-dose and prolonged methotrexate therapy^15^

Surgical options that preserve fertility have been tried. Cerclage is not currently recommended. Suction curettage can be performed with subsequent Foley catheter placement in the cervix to tamponade bleeding. Uterine artery embolization can minimize intraoperative bleeding.

Historically, hysterectomy was often the only treatment option, which stopped the profuse hemorrhage that accompanies attempts at removal of a cervical pregnancy. Even today, surgery is chosen either as a last resort when medical management fails, or in emergency situations when a woman, usually previously undiagnosed, presents with life-threatening acute hemorrhage.^15^ A cervical pregnancy that is undetected until the second or third trimester usually requires a hysterectomy.^11^ This was unfortunately the case in our patient, who had an undiagnosed, second-trimester, cervical pregnancy that was already ruptured at presentation. Because of her ongoing hemorrhage, conservative measures were not an option for her; she needed a total hysterectomy to control the hemorrhage.

## CONCLUSION

In summary, cervical pregnancy is a rare but potentially fatal form of ectopic pregnancy. An emergency physician must be aware of the possibility, and combine a high index of suspicion with clinical and radiological findings to make an accurate and timely diagnosis of cervical pregnancy. Ultrasound is the most important diagnostic tool in early detection of cervical pregnancy, although MRI can be useful if ultrasound is not definitive. Though medical management is preferred, its success depends directly on how early the diagnosis is made. Failure of conservative management may necessitate more aggressive surgical intervention and even a total hysterectomy to control the hemorrhage.

Documented patient informed consent and/or Institutional Review Board approval has been obtained and filed for publication of this case report.
